# Transcriptome and Endogenous Hormones Reveal the Regulatory Mechanism of Flower Development in *Camellia azalea*

**DOI:** 10.3390/plants14152291

**Published:** 2025-07-25

**Authors:** Jian Xu, Fan Yang, Ruimin Nie, Wanyue Zhao, Fang Geng, Longqing Chen

**Affiliations:** Yunnan Engineering Research Center for Functional Flower Resources and Industrialization, College of Landscape and Horticulture Sciences, Southwest Forestry University, Kunming 650224, China; xujian960128@163.com (J.X.); yf@swfu.edu.cn (F.Y.); nieruimin1313@163.com (R.N.); zhaowanyue0112@163.com (W.Z.); fanggeng20@swfu.edu.cn (F.G.)

**Keywords:** *Camellia azalea*, transcriptome sequencing, differential expressed genes (DEGs), floral development, endogenous hormones

## Abstract

*Camellia azalea* is an endemic species within the genus *Camellia* that exhibits the trait of summer flowering, which is of significant ornamental and research value. Nevertheless, research on the regulatory mechanisms of flower formation in *C. azalea* is still limited, so in this study, transcriptome sequencing and analysis of endogenous hormone contents were conducted at three distinct growth stages: floral induction, floral organ maturation, and anthesis. Illumina sequencing yielded a total of 20,643 high-quality unigenes. Comparative analyses of representative samples from the three growth stages identified 6681, 1925, and 8400 differentially expressed genes (DEGs), respectively. These DEGs were further analyzed for functional enrichment using the GO and KEGG databases. Additionally, core genes from each flowering pathway underwent expression pattern analysis and network diagram construction. This revealed that the flower development process in *C. azalea* is linked to the specific expression of the genes involved in the photoperiod, temperature, and autonomous pathways and is subject to comprehensive regulation by multiple pathways. Further analysis of the dynamic trends of five endogenous hormone contents and plant hormone signal transduction genes revealed significant differences in the requirements of endogenous hormones, such as gibberellins and indoleacetic acid, by *C. azalea* at distinct growth stages. Additionally, the majority of genes on the phytohormone signal transduction pathway demonstrated a high correlation with the changes in the contents of each hormone. The present study integrates physiological and molecular approaches to identify key genes and metabolic pathways that regulate the summer flowering of *C. azalea*, thereby laying a theoretical foundation for further investigations into its flowering mechanism and related functional genes.

## 1. Introduction

Over an extended period of evolution, higher plants have evolved a sophisticated and extensive regulatory mechanism for flower formation in vivo. This mechanism is regulated by a combination of genetic factors, endogenous hormones, and external environmental conditions [1,2]. The current body of research indicates that the flowering mechanism of model plants, such as *Arabidopsis thaliana* and *Antirrhinum majus*, can be regulated by external signals, including light and temperature, in response to the photoperiodic, vernalization, and temperature pathways. Additionally, the autonomous, gibberellin, and age pathways can promote flower formation by sensing endogenous signals [3,4,5]. Subsequently, these pathways act on flowering integration genes, including *Flowering Locus T* (*FT*), *Suppressor of Overexpression of Constans 1* (*SOC1*), and *Flowering Locus C* (*FLC*), to activate floral meristem-specific genes, such as *Apetala 1* (*AP1*), *Leafy* (*LFY*), and *Agamous* (*AG*), thereby regulating the flowering time of plants [6,7,8,9]. The publication of the complete genome sequence of the cash crop tea (*Camellia sinensis*) has provided a robust theoretical framework for investigating the mechanisms underlying flower formation in *Camellia* [10]. Relevant studies have demonstrated that the differentiation in floral genes during the flowering process of *Camellia* has significant biological implications. For instance, the dormancy-related MADS-box genes, *Agamous-like* (*AGL*) and *Short Vegetative Phase* (*SVP*), are expressed at different stages of flower bud differentiation in *C. azalea*, which is regulated by multiple pathways, including those responsive to low temperatures and light signals [11]. Furthermore, it was hypothesized that the *CaACO1* gene might influence flower development and floral organ senescence in *C. azalea* based on its correlation with morphological flower indicators [12]. *Camellia perpetua* exhibits a distinctive flowering mechanism compared to *Camellia petelotii*, characterized by the pronounced upregulation of the flower-forming hormone gene *FT*, along with the flowering integration genes *SOC1* and *AP1* [13].

Flowering in plants is regulated not only by external environmental factors but also by endogenous hormone signals throughout the entire growth and development process. Hormonal signaling is regulated by the detection of environmental changes, integration and transduction of various hormonal signals, and modification of expression levels of key genes involved in flower formation, aiming to promote flowering [14,15]. Gibberellins (GA) play a pivotal role in the formation of flowers in plants. Inhibition of GA synthesis or disruption of its signaling affects flower formation in *A. thaliana*, and conversely, induces the expression of the flower-forming genes *FT*, *SOC1*, and *LFY* to promote flowering [16,17]. Indoleacetic acid (IAA) regulates a variety of life processes, including plant cell differentiation and embryo development. IAA is also a key factor affecting flower formation in plants [18]. Notably, the regulatory effects of IAA on flower formation vary considerably among different species. For instance, high levels of IAA promoted flower formation in *Vitis vinifera*, whereas low levels of IAA favored bud differentiation and flowering in *C. perpetua* [19,20]. In addition, it has been reported that differences in IAA levels regulate the expression of a variety of genes associated with GA metabolism, which in turn affects plant flower formation by regulating GA synthesis and transduction [21]. Recent research has also focused on other endogenous hormones, including abscisic acid (ABA), cytokinin (CTK), and brassinosteroid (BR), and their roles in regulating flower formation in plants [22,23,24]. In summary, multiple endogenous hormones operate independently of one another and interact with each other, forming a comprehensive genetic signaling network of synergistic and antagonistic coexistence in plants.

*C. azalea* is an evergreen shrub or small tree, which belongs to the family Theaceae. It has a wide range of potential applications in ornamental and production horticulture due to its notable characteristics, including large and colorful flowers, a long flowering period, and strong resistance to adversity [25,26]. Unlike most Camellias, which bloom in winter and spring, *C. azalea* distinctly flowers in summer and even into autumn. Under optimal conditions, it can achieve continuous flowering across all seasons [27,28]. In comparison to the winter–spring flowering Camellia, *C. azalea* exhibits notable distinctions in its growth and developmental characteristics. The regulatory mechanisms that underlie flower formation are still poorly understood. Moreover, previous studies on *C. azalea* have predominantly focused on its morphological, physiological, and biochemical characteristics. Although the gene expression profiles of *C. azalea* at different stages of floral development have been reported in the literature and the floral transition has been discussed, the key factors affecting the flowering of *C. azalea* have not been investigated in the context of the changes in endogenous hormone content [29]. In this study, we sequenced the transcriptomes of *C. azalea* from various developmental stages, aiming to identify key genes that play significant regulatory roles in each period. Additionally, the dynamic trends in endogenous hormones and related gene expression across different developmental periods were also studied, initially exploring the key factors influencing flower formation in *C. azalea*. The objective was to identify the key genes with important regulatory roles at each developmental stage.

## 2. Results

### 2.1. Observation and Sampling of C. azalea at Different Developmental Stages

**Figure 1:**
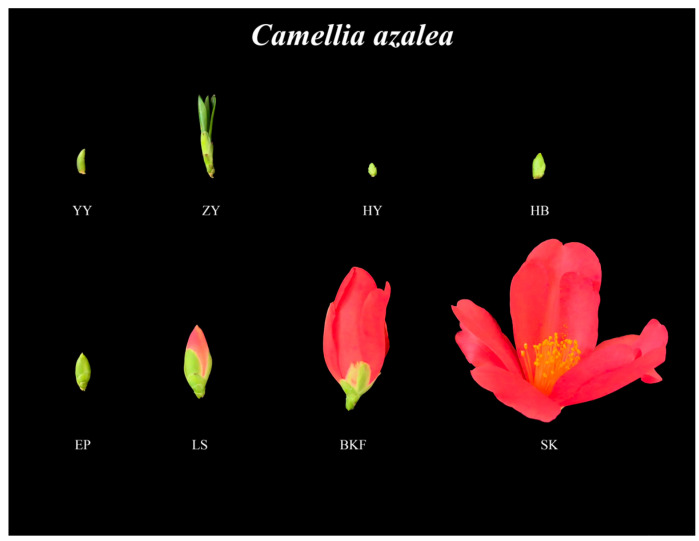
Different flower development stages of *C. azalea*. During the vegetative growth stage, two developmental stages were collected: the leaf bud stage (YY) and the leaf expansion stage (ZY). During the reproductive growth stage, six developmental stages were collected: the flower bud formation stage (HY), the flower bud stage (HB), the sepal stage (EP), the color showing stage (LS), the early flowering stage (BKF), and the full bloom stage (SK).

This study observed the growth and development of *C. azalea* and collected sample materials to analyze the dynamic changes in endogenous hormones and performed transcriptome sequencing across its different developmental stages (Figure 1). Specifically, a total of 2 developmental stages were sampled during the vegetative growth period, namely the leaf bud stage (YY) and leaf expansion stage (ZY). After maturation of current-year young shoots, flower buds germinate in clusters around the branch apical meristem, and *C. azalea* subsequently enters the reproductive growth stage. During the reproductive growth period, a total of 6 developmental stages were sampled, namely flower bud formation stage (HY), flower bud stage (HB), sepal stage (EP), color exposure stage (LS), early flowering stage (CK), and full bloom stage (SK).

### 2.2. Statistical and Functional Annotation of Transcriptome Sequencing Data

Transcriptome sequencing of RNA samples from the different developmental periods of *C. azalea* was performed. The study’s results showed that the raw data from the 24 sample materials ranged between 358,564,380 and 461,196,440 entries. The total number of bases ranged from 54,143,221,380 to 69,640,662,440 base pairs. The percentage of Q30 bases was 93.48% and above. Following the removal of low-quality reads, the percentage of high-quality sequence reads to sequenced reads and the percentage of high-quality sequence bases to sequenced bases were both greater than 93.8%. This resulted in the identification of 20,643 unigenes (Table S1). In order to gain a comprehensive understanding of the bioinformatic gene functional information of *C. azalea*, the obtained unigenes were subjected to functional annotation in six public databases: Kyoto Encyclopedia of Genes and Genomes (KEGG), Gene Ontology (GO), Protein Family Database (PFAM), Swiss-Prot, evolutionary genealogy of genes: Non-supervised Orthologous Groups (eggNOG), and NCBI non-redundant protein sequences (NR). The number of unigenes annotated to the NR database was 20,079 (97.27%), while only 7975 (38.63%) unigenes were annotated to the KEGG database with respect to biological functions. A total of 5684 (27.53%) unigenes were annotated with functional information in all six databases (Figure S1A). As one of the most comprehensive public protein databases, NR contains a vast number of protein sequences with known functions. Homology analysis of unigenes against sequences in the NR database allows for the rapid inference of the potential functions of proteins encoded by these unigenes. The results of the comparison of the NR database revealed that *C. azalea* had 17,683 unigenes with a high degree of homology with *C. sinensis* in terms of species homology, which accounted for 88.07% of the total number of unigenes (Figure S1B). Regarding sequence matching similarity, the most common similarity interval was 95–100% (69.81%), with only 122 sequences showing similarity below 40% (0.61%). In conclusion, *C. azalea* exhibits a high degree of similarity to the NR database and is closely related to *C. sinensis* (Figure S1C).

### 2.3. C. azalea Sample Correlation Analysis

**Figure 2:**
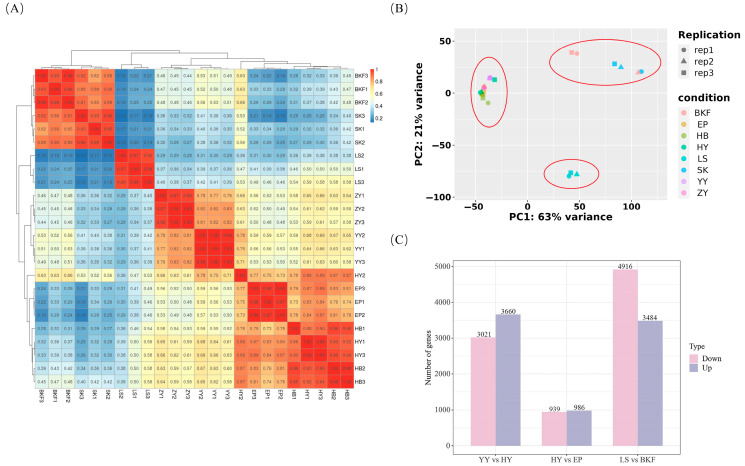
*C. azalea* sample correlation analysis and DEG screening: (**A**) Correlation analysis of sequencing samples. The left and top sides of the figure show the sample clustering situation, the right and bottom sides show the sample names, and the different colored squares represent the high and low correlation situation of the two samples. (**B**) Sequencing sample clustering PCA. The horizontal coordinate is the first principal component, and the vertical coordinate is the second principal component. Different shapes in the figure indicate different samples, and different colors indicate different groupings. (**C**) Screening of DEGs at different growth stages, including YY vs. HY, HY vs. EP, and LS vs. BKF.

In this experiment, Pearson’s correlation coefficient was employed to quantify the correlation between gene expression levels across samples. The correlation between all the biological replicates of *C. azalea* collected during the same period was found to be greater than 0.8, indicating a high degree of correlation between each group of replicates. Figure 2A indicates that the two periods of *C. azalea* nutritional stage, YY and ZY, were clustered into one group to a greater extent than the first three periods of reproductive stage, HB, HY, and EP, which were divided into one group. Furthermore, the correlation coefficients between their two developmental periods were all above 0.7. It is noteworthy that the three biological replicates in the LS period were grouped separately, despite being classified as part of a larger group with the previous periods. This suggests that *C. azalea* exhibited a more pronounced difference in gene expression during this period. Additionally, the BKF and SK periods showed lower correlations with other periods and were categorized distinctly (Figure 2A). Meanwhile, the results of a PCA (principal component analysis) were consistent with the results obtained from a correlation analysis (Figure 2B).

### 2.4. DEG Screening and Enrichment Analysis

This experiment identified three critical stages in the growth and development of *C. azalea* based on the observations of the external morphology of *C. azalea* and the results of inter-sample correlation analysis. The first stage was floral induction, which corresponds to *C. azalea* entering the reproductive growth stage around April. However, the emergence of flower buds and leaf buds could be observed at the same time in branches at different locations. Therefore, the sample database of these two periods was selected for comparative analysis with a view to exploring the differences in the mechanisms of growth and development of leaf buds and flower buds in *C. azalea*. The second stage was floral organ maturation, which involves the initiation and cessation of differentiation of the majority of floral organs. *C. azalea* initiated the differentiation of the majority of floral organs at the HY, which persisted until the EP. At this point, the growth of each differentiation primordium ceased, marking the end of the floral bud differentiation process. The third stage was anthesis, during which *C. azalea* buds were fully developed before opening, and then flowered a few days later. In addition, the results of this stage delineation are consistent with the clustering results obtained from the sample correlation analysis (Figure 2A), further demonstrating the importance of the three stages mentioned above.

Using YY as the control, 6681 DEGs were identified in HY, with 3660 significantly upregulated and 3021 significantly downregulated. A total of 1925 DEGs were screened in floral organ maturation (using HY as the control). Of these, 986 were significantly upregulated, while 939 were significantly downregulated in expression. In anthesis (using LS as the control), 8400 DEGs were identified, with 3484 and 4916 genes exhibiting significant upregulation and downregulation, respectively (Figure 2C).

**Figure 3:**
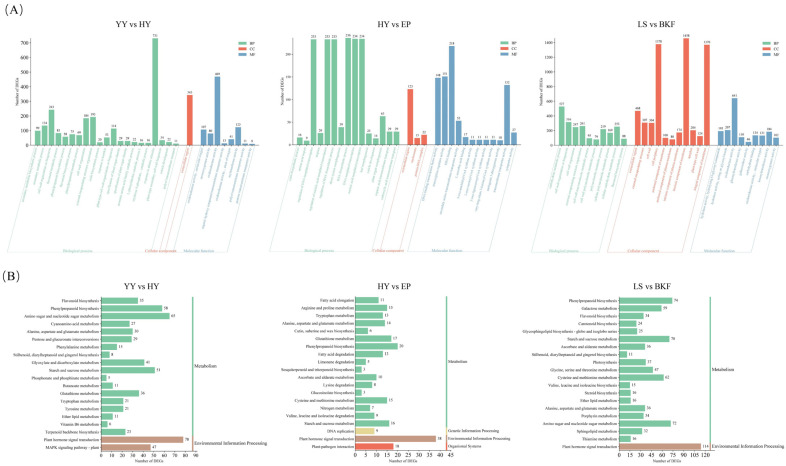
Functional enrichment analysis of *C. azalea* DEGs: (**A**) GO enrichment analysis of DEGs at different growth stages of *C. azalea*. The horizontal coordinate is the GO term, and the vertical coordinate is the number of enriched DEGs. (**B**) KEGG enrichment analysis of DEGs at different growth stages of *C. azalea*. The horizontal coordinate is the number of enriched DEGs, and the vertical coordinate is the pathway.

Furthermore, we investigated the differences in gene function across various growth stages of *C. azalea* (Figure 3A). To this end, the DEGs obtained from each of the three growth stages were annotated and subjected to analysis in the Gene Ontology (GO) database. During floral induction, most DEGs were significantly enriched in response to abiotic stimuli (731 DEGs) and oxidoreductase activity (469 DEGs). A total of 29 DEGs with the specification of plant organ identity were found to be upregulated. Further annotation and analysis of the DEGs in this pathway revealed that they were primarily classified into three families: YABBY, MADS-box, and WUSCHEL-related homeobox (WOX), with the vast majority belonging to MADS-box genes (Supplementary Table S2). During floral organ maturation, the most significantly enriched terms at this growth stage were primarily those related to plant organ morphogenesis (63 DEGs) and regulation of the RNA biosynthesis process (233 DEGs). Finally, during anthesis, significantly enriched terms included carbohydrate metabolic processes (527 DEGs), cell wall organization or biogenesis (316 DEGs), and intrinsic components of the membrane (1458 DEGs).

The KEGG enrichment analysis showed that the top 20 pathways significantly enriched at each key stage were predominantly related to metabolism. These pathways included phenylpropanoid biosynthesis (ko00940), starch and sucrose metabolism (ko00500), and amino sugar and nucleotide sugar metabolism (ko00520), among others, which were significantly enriched in more than 50 DEGs. Meanwhile, the plant hormone signal transduction (ko04075) under the broad category of environmental information processing was significantly enriched in each growth stage and contained most DEGs, and the number of DEGs enriched in each pathway could also indirectly indicate where the differences in life activities were found in different growth stages. Furthermore, *C. azalea* exhibited a notable enrichment in pathways associated with photosynthesis (ko00195)and carotenoid biosynthesis (ko00906) during anthesis (Figure 3B).

### 2.5. Cluster Analysis of DEGs During the Flowering Development Process in C. azalea

**Figure 4:**
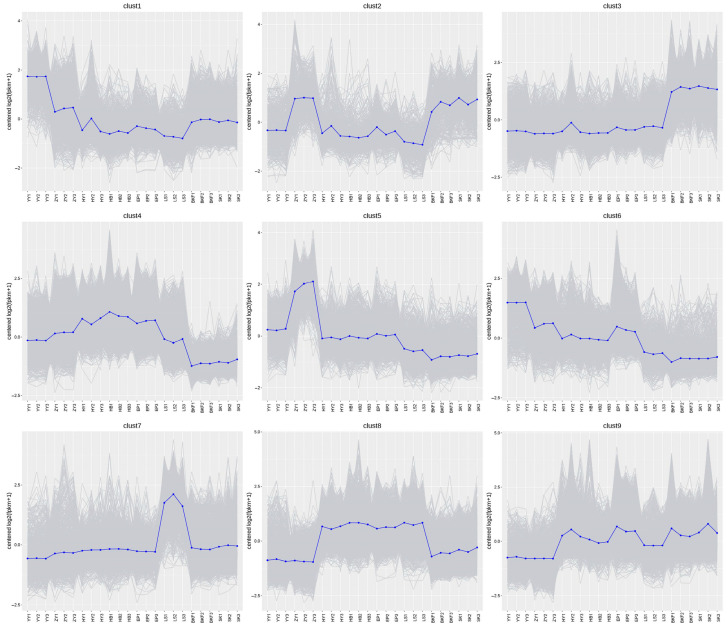
Expression clustering analysis of *C. azalea* DEGs. The gray line in the figure shows the expression pattern of the genes in each cluster, and the blue line shows the average expression of all the genes in the cluster in the sample.

In order to gain insight into the dynamic trends of differential genes during the development of the *C. azalea* flower, a total of 17,216 differential genes were co-clustered into nine gene clusters based on the K-Means method (Figure 4). In conjunction with the period of growth and development, at the stage of flower induction (YY vs. HY), the expression of genes belonging to Cluster 1 and Cluster 6 decreased significantly at the HY in comparison to the YY. In contrast, the expression levels of Cluster 4 and Cluster 9 genes increased significantly, indicating that these genes play distinct roles in the process of flower induction. The expression of Cluster 8 genes remained at a high level throughout the floral organ maturation stage, before significantly declining upon entering anthesis, indicating a positive regulatory effect on the development of *C. azalea* floral organs. Clusters 3 and 7 comprised genes specifically expressed before and after anthesis. Cluster 3 was expressed at a low and stable level during the first few developmental periods until anthesis (BKF~SK), when it showed a large increase and remained at a high expression level. In contrast, the Cluster 7 gene had a very high level of transcripts only in the LS, and its expression was not significantly different in the rest of the developmental periods. This indicates that the growth of organisms at different developmental stages is closely associated with specific gene clusters.

### 2.6. Expression Pattern Analysis of Key Genes for Flower Formation

**Figure 5:**
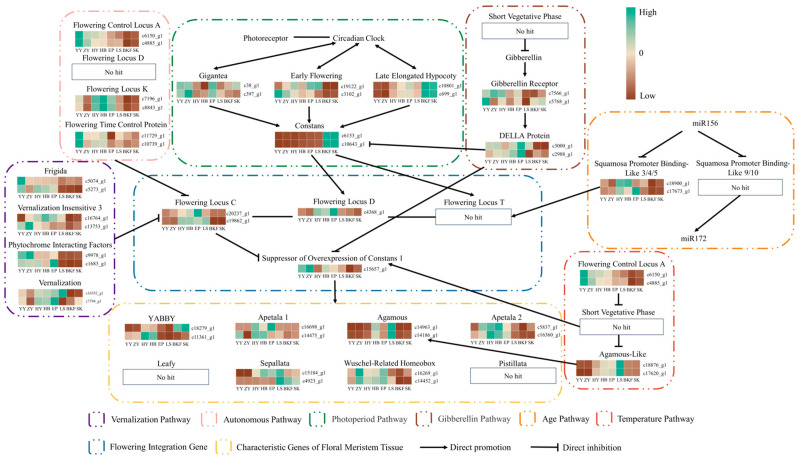
Regulatory network diagram of the floral pathway in *C. azalea*. Expression heatmap based on transcriptome sequencing data using the FPKM values of some key genes screened, with high expression in green and low expression in brown. Arrows indicate promotion, and horizontal lines indicate repression. Different colored dashed boxes indicate different pathways or types of key genes.

We selected several genes related to the flower formation pathway, as identified in the relevant literature, to analyze expression patterns and construct network diagrams (Figure 5). The results showed that core genes in the vernalization pathway, *FRIGIDA* (*FRI*), *VERNALIZATION INSENSITIVE 3* (*VIN3*), *PHYTOCHROME INSENSITIVE FACTOR* (*PIF*), and *VERNALIZATION* (*VRN*), exhibited relatively high expression levels to varying degrees during floral induction (YY~HY) and floral organ maturation (HY~EP), whereas their expression decreased significantly during anthesis. In the autonomous pathway, flowering-promoting genes *FLOWERING LOCUS CA* (*FCA*) and *FLOWERING LOCUS PA* (*FPA*) exhibited the highest expression during YY, with decreased expression levels in other stages. Conversely, *FLOWERING LOCUS K* (*FLK*) was significantly upregulated during floral organ maturation (HY~EP). All the aforementioned genes can negatively regulate the expression of the flowering repressor gene *FLC*, thereby promoting plant flowering. However, the *FLC* gene in *C. azalea* did not exhibit a generally opposite expression pattern to these genes. The circadian rhythm genes *GIGANTEA* (*GI)* and *EARLY FLOWERING* (*ELF*) in the photoperiod pathway exhibited increased expression primarily during YY, ZY, and LS, followed by significant downregulation at the BKF. Conversely, the *LATE ELONGATED HYPOCOTY* (*LHY*) gene maintained a low expression level during the early developmental stages of *C. azalea*, and its expression level did not peak until anthesis (BKF~SK). The *CONSTANS* (*CO*) gene promotes flowering by integrating photoperiod and circadian rhythmicity signals to activate the expression of downstream genes. Its expression pattern is similar to that of the *LHY* gene, showing a relatively significant upregulation during anthesis (BKF~SK). The expression levels of gibberellin receptor genes (gibberellin pathway) were relatively high during YY~HY, while decreasing to the lowest levels during LS~BKF. The negative regulator, the DELLA protein, gradually increased in expression during floral organ maturation (HY~EP) and then significantly decreased during LS. The DELLA protein also exerts a repressive effect on the expression of *CO* genes. In the temperature pathway, the *FCA* gene exhibited the highest expression level in *C. azalea* at the YY, and then gradually decreased in expression level. The *AGL* gene exhibited an expression trend of first increasing and then decreasing, with its highest expression level observed at the LS. In the age pathway, only the *SPL3/4/5* genes were identified, and their expression gradually increased during floral organ maturation (HY~EP). The flowering integration genes primarily included *FLC*, *FD*, *FT*, and *SOC1*. Of these, *FLC* and *FD* genes exhibited relatively significant upregulated expression during floral organ maturation (HY~EP), whereas the *SOC1* genes, which promote floral bud differentiation, were more highly expressed at YY. The core genes in these floral pathways act together on floral integrator genes (e.g., *FT*, *SOC1*, and *FLC*), thereby activating inflorescence meristem-specific genes such as *YABBY*, *Apetala1*, *Apetala2*, and *WOX*. These genes typically exhibit high expression during HY~EP to promote the differentiation and development of floral organs in *C. azalea*.

### 2.7. Expression Pattern Analysis of Key Genes Involved in Plant Hormone Signal Transduction

**Figure 6:**
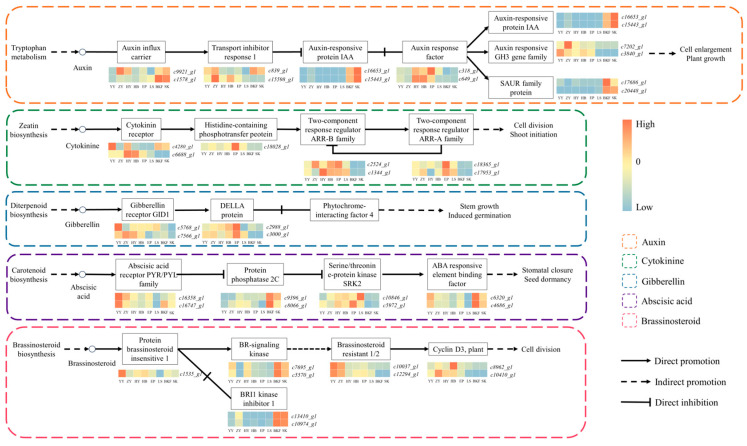
Diagram of the regulatory network of the plant hormone signal transduction pathway. Some key genes on the endogenous IAA, CTK, GA, ABA, and BR signaling pathways were screened, and the expression heat map was performed using the FPKM values of transcriptome sequencing data, with high expression in red and low expression in blue. Solid arrows indicate direct facilitation, dashed arrows indicate indirect facilitation, and solid horizontal lines indicate direct inhibition. Different colored dashed boxes indicate key genes on different endogenous hormone signaling pathways.

We analyzed the transcriptome sequencing data of *C. azalea* to identify core genes from the plant hormone signal transduction pathway for expression pattern analysis (Figure 6). The figure shows that AUXIN RESPONSE FACTOR (ARF) exhibited a relatively high expression level only during floral organ maturation (HY~EP), while AUXIN-RESPONSIVE PROTEIN (Aux/IAA) and SAUR family proteins exhibited a significant increase in expression during anthesis (BKF~SK). In the cytokinin signaling pathway, the expression levels of the TWO-COMPONENT RESPONSE REGULATOR ARR family (ARR) mostly peaked at HY or EP, whereas they were barely expressed after entering anthesis (BKF~SK). These genes may play an important regulatory role in the differentiation and maturation of *C. azalea* floral organs. In the gibberellin signaling pathway, GIBBERELLIN RECEPTOR genes exhibit enhanced expression during YY~HY, and then gradually weaken. DELLA proteins exhibit a general pattern of initial increase followed by a decrease, with their expression peaking at the EP and starting to decline at the LS. From the expression heatmap of genes related to the abscisic acid signaling pathway, it can be observed that ABA RECEPTOR genes exhibit enhanced expression exclusively during YY. PROTEIN PHOSPHATASE genes and ABA-RESPONSIVE ELEMENT BINDING FACTOR (ABF) display a similar expression pattern, with both being significantly upregulated during anthesis (BKF~SK). In the brassinosteroid signaling pathway, BRASSINOSTEROID RESISTANT 1/2 exhibits high expression levels during YY~ZY, and their expression levels then gradually decrease in the HY~SK. In contrast, BR- SIGNALING KINASE and BRI1 KINASE INHIBITOR 1 display an expression pattern of initially low levels followed by an increase, with their transcriptional levels peaking at the BKF. Comprehensive analyses demonstrate that distinct genes within the same hormone signal transduction pathway display dynamic differential expression patterns across different growth stages, suggesting that these pathway-specific genes exert specific biological functions at particular growth stages and collectively contribute to the growth and development of *C. azalea*.

### 2.8. Determination of Endogenous Hormone Content in C. azalea During Development

**Figure 7:**
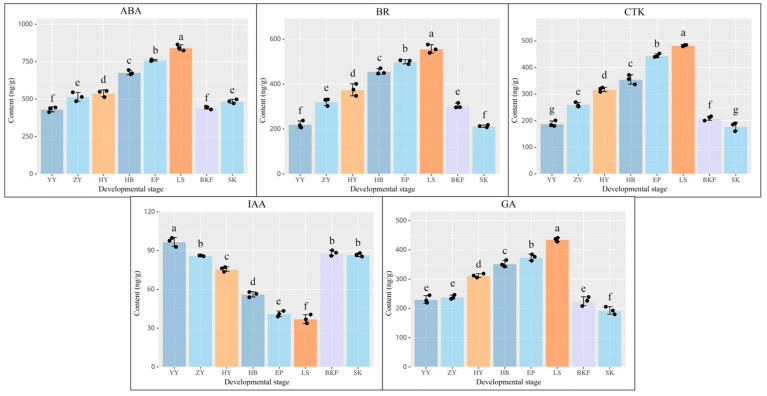
*C. azalea* endogenous hormone determination at different developmental stages. Endogenous ABA, BR, CTK, IAA, and GA content trends were determined for different developmental stages in *C. azalea*. The horizontal coordinates represent the eight developmental stages; the vertical coordinates represent the endogenous hormone content. The data were analyzed in three biological replicates, and standard deviations were shown with error bars. Different lowercase letters in the figure indicate significant differences.

Figure 7 illustrates changes in the GA, ABA, IAA, CTK, and BR content during various developmental periods of *C. azalea* (Figure 7). Throughout the developmental phases, all five endogenous hormones exhibited significant differences (*p* < 0.01) between adjacent periods. Among the substances in question, GA, ABA, CTK, and BR were maintained at low concentration levels during the nutritional stage (YY and ZY). Their contents steadily increased during the pre-reproductive stage, peaking at LS as the developmental process progressed. After that, a drastic downward trend was observed. Conversely, IAA exhibited a pattern of declining and subsequently rising throughout development, with the highest IAA content observed at YY (96.70 ng/g) and the lowest at LS (37.04 ng/g). A comprehensive analysis revealed that the contents of the five endogenous hormones displayed a stable and consistent trend during the growth and development of *C. azalea*, peaking at LS. Therefore, it can be hypothesized that there is a significant differentiation between the plants themselves at LS and other developmental periods, with large changes in their internal transcriptional profiles. These changes may promote normal flowering in *C. azalea.*

### 2.9. Analysis of Endogenous Hormones and Key Gene Correlations

The correlation between the levels of five endogenous hormones and the expression of individual hormone-related genes in different developmental periods (Figure S2). The results showed that changes in the content of ABA, BR, and GA were negatively correlated to varying degrees with the expression of most of the majority of their associated genes, whereas the majority of genes on the CTK pathway exhibited a positive correlation with changes in content. It is noteworthy that on the IAA pathway, the majority of the ARF genes showed a significant negative correlation with IAA content. In addition, other types of genes may demonstrate both positive and negative correlations in regulating the changes in IAA content depending on their respective functions.

### 2.10. qRT-PCR Validation

**Figure 8:**
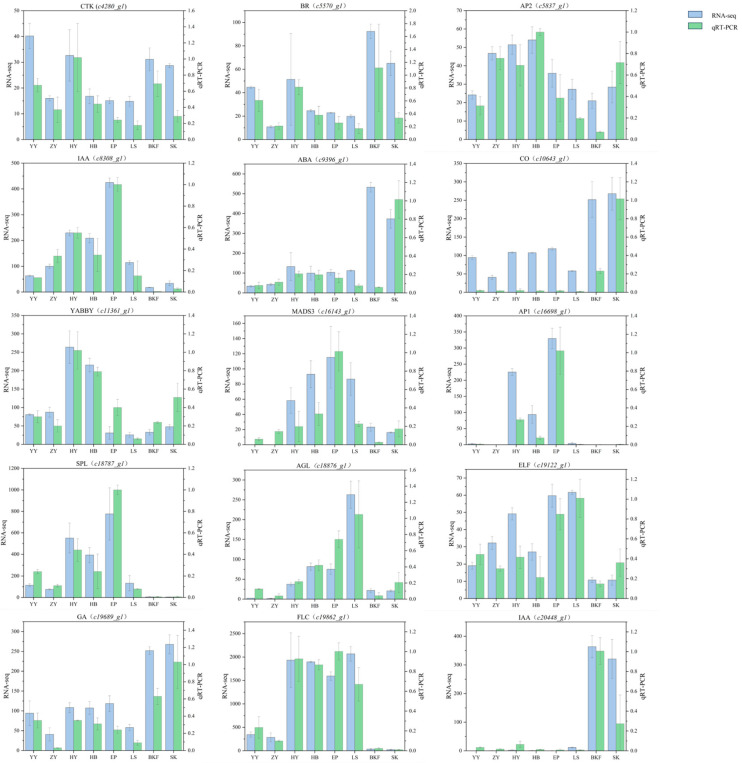
Results of the qRT-PCR validation of the sequencing data. The expression levels of 15 flower development-related genes at different developmental periods were verified by qRT-PCR. The horizontal coordinates are the eight developmental periods, the vertical coordinates are the gene expression levels, the blue bars are the sequencing data, and the green bars are the quantitative data. The data were analyzed in three biological replicates, and standard deviations were shown with error bars.

To verify the accuracy of gene expression results from pre-transcriptome sequencing data, 15 differentially expressed genes were selected for quantitative real-time PCR validation. These genes were selected from those flower meristem characterization genes, the floral pathway, and the genes related to the plant hormone signal transduction pathway, respectively. The validation results of the sequencing data are presented in Figure 8. The relative expression of the selected genes demonstrated a high degree of correlation with the expression change trends observed in the transcriptome sequencing data. Additionally, the results provide further evidence that the sequencing accurately reflected the transcript changes in *C. azalea* across various developmental stages.

## 3. Discussion

### 3.1. Sample Correlation and PCA

Analysis of 24 sequenced samples from eight developmental periods of *C. azalea* revealed high correlation and reproducibility among various biological replicates, confirming the accuracy of the sequencing data (Figure 3A,B). The cluster analysis revealed that the process of *C. azalea* flower development can be broadly divided into three growth stages: flower induction, floral organ maturation, and anthesis. Flower induction is a pivotal phase in plant growth, marking the transition from leaf buds to flower buds. The DEGs between YY and HY offer further insights into the induction of reproductive growth in *C. azalea*. The three developmental stages HY~EP in the floral organ maturation stage showed a high correlation, and the DEGs in this stage may contain regulatory factors that affect floral organ maturation. Sample material from anthesis (BKF~SK) differed significantly from previous developmental periods and was thus categorized separately, indicating considerable changes in the transcriptional profiles of internal genes as *C. azalea* buds develop and mature into flowers. Therefore, through the screening of differential genes and functional enrichment analysis across the three main developmental stages, it is possible to systematically dissect and accurately identify the key genes and signaling pathways that regulate the summer flowering of *C. azalea*.

### 3.2. Identification and Functional Annotation of DEGs at Different Growth Stages of C. azalea

The process of flower development is the result of the interaction of multiple biosynthetic, transduction, and metabolic pathways. DEGs at the stage of flower induction were significantly enriched in response to abiotic stimuli, oxidoreductase activity, and plant organ morphogenesis. (Figure 3A) DEGs enriched by the plant organ identity specification pathway are mainly classified into three families, YABBY, MADS-box, and WOX. The YABBY gene primarily regulates the initiation of apical meristematic tissue (SAM) development in embryos and the formation of floral organs [30,31]. MADS-box genes, which function as transcription factors and may be involved in flower development, play a pivotal role in the development of organs such as petals, stamens, carpels, and seeds, among others. Although these genes belong to more detailed branches, respectively, it can be speculated that there is a common regulatory mechanism, which is worthy of further studies [32]. The role of WOX genes in regulating plant cell division and proliferation, as well as the tracheal system and embryonic development, is of significant biological importance [33]. Following this, it was hypothesized that these DEGs mentioned above are closely linked to the induction of flower formation in *C. azalea*. After flower buds have formed, the primordia of each floral organ are initiated, and the specific expression of some genes in the plant promotes the maturation of the floral organs. Significantly enriched pathways at this stage include plant organ morphogenesis and the regulation of the RNA biosynthesis process (Figure 3A). Plants respond to internal and external environmental signals, and RNA controls the maturation of the plant’s various floral organs by regulating the transfer of genetic information and protein synthesis through replication and transcription. The major pathways for the significant enrichment of DEGs at anthesis include carbohydrate metabolism, cell wall organization or biogenesis, and cell wall macromolecular metabolic processes (Figure 3A). The literature demonstrates that cell wall formation is crucial in determining the size and morphology of floral organs. In addition, cell walls can promote intercellular transport and transfer of substances to meet the needs of normal growth and development of the plant body, thus promoting flowering and improving the plant’s resistance to stress [34,35].

The KEGG pathway analyses revealed some overall similarity in the pathways enriched by DEGs in the three growth stages of *C. azalea*. Pathways such as plant hormone signal transduction, phenylpropanoid biosynthesis, and starch and sucrose metabolism were significantly enriched in all three growth stages, suggesting that there are significant differences in endogenous hormones and other substances and energy requirements in different growth stages of *C. azalea* (Figure 3B). In contrast, pathways such as tryptophan, arginine, and proline metabolism were significantly enriched during the floral organ maturation stage, suggesting that the metabolism of these amino acids plays a crucial role in the early differentiation of floral organs in *C. azalea*. For example, tryptophan is not only a precursor of growth hormone, which can effectively regulate plant growth and development, but also plays a crucial role in photosynthesis and improving plant resistance [36,37]. After entering anthesis, genes enriched in pathways such as photosynthesis and carotenoids are differentially expressed, and the increased efficiency of photosynthesis may be effective in promoting the synthesis and decomposition of organic substances such as carbohydrates and secondary metabolites. These substances not only promote the establishment of plant morphology and structure but also regulate physiological and biochemical processes, thus supporting the plant’s growth and development and providing the energy needed for its vital functions [38]. Thus, the process of flower formation in *C. azalea* is influenced by a variety of pathways and metabolites, and these pathways, which were significantly enriched as described above, may be biologically important at different growth stages.

### 3.3. The Role of Various Floral Pathways in the Development of the Flower of C. azalea

Referencing six major flowering pathways in model plants, we selected several core genes and analyzed their expression patterns to explore the potential regulatory network of flowering in *C. azalea* (Figure 6). The photoperiod pathway, a key determinant of flowering in plants, directly influences plant growth, development, and morphogenesis. In *C. azalea*, the upstream genes in the photoperiodic pathway, *GI* and *ELF*, were highly expressed during the early stages of growth and development, suggesting that these genes may play a role in flower induction or floral organ maturation. However, the expression of the late-flowering gene *LHY* was only significantly increased at anthesis, which is consistent with the findings that the highest expression of the *ImLHY* gene in Impatiens morsei is at anthesis [39]. CO proteins regulate stable accumulation through light signaling and the circadian clock, inducing the expression of the florigenin *FT* gene. *FT* proteins are then translocated through the phloem from the leaf to the stem’s tip, forming a complex with *FD*, which activates the expression of genes such as *SOC1* [40,41]. In this study, the expression of the CO genes increased dramatically upon entering anthesis, while the *FD* genes had the highest expression during the floral organ maturation stage, whereas the *FT* genes were not identified. Plant leaves are the key site for sensing photoperiodic changes, and genes such as CO and *FT* are mainly expressed in leaves, whereas the materials used in this study did not include mature leaves. Therefore, further investigation into the photoperiod pathway’s effects on *C. azalea* flowering should include analysis of these genes’ expression patterns in leaves across various developmental stages [42]. The level of external ambient temperature has a significant effect on the flowering time of plants. The upstream genes, such as *FRI*, *VRN,* and *FCA*, were highly expressed to varying degrees during the early stages of development, but were maintained at lower expression levels after entering anthesis, and it can be speculated that these upstream genes generally function to promote flower bud maturation during the predevelopment period. In contrast, the *FLC* gene showed a similar expression pattern, with significant downregulation during the anthesis of *C. azalea*. The *FLC* gene is a key regulator of flowering repression in Arabidopsis and acts directly on the CArG structural domains of *SOC1*, *FD,* and *FT* to repress their expression and consequently flower formation [43]. However, *C. azalea* flowers at high summer temperatures, and correspondingly, *FCA* exhibits higher transcript and protein levels under these conditions [44]. Therefore, it can be hypothesized that during the development of *C. azalea*, *FLC* genes may be more affected by genes located upstream of the autonomous pathway or the temperature pathway, such as *FCA*, *FLK,* and *FPA*, and thus involved in the regulation of flower formation. Located in the age pathway, *SPL* genes are plant-specific transcription factors [45]. In A. thaliana, *SPL3/4/5* were found to mainly promote the development of floral meristematic tissues, whereas *SPL9/10* induced the transition from nutrient to reproductive growth [46]. Following the formation of *C. azalea* flower buds, the expression of *SPL3/4/5* genes gradually increased until the flower buds fully developed, then significantly decreased, suggesting a promotional effect on floral organ differentiation. However, *SPL9/10* genes were not identified, possibly because these genes had already acted to promote flower bud formation before the sampling at the bud stage.

### 3.4. Effects of Plant Hormones on Flower Formation in C. azalea

The gibberellin pathway is one of the earliest demonstrated pathways of flower formation and is bound to play a key role in plant flower formation. Gibberellin signaling is a plant response to gibberellin signals, which mediates the degradation of DELLA proteins through the gibberellin receptor *GID1* gene, thus regulating flower formation [14]. The DELLA protein gene in *C. azalea* showed an expression pattern of first increasing and then decreasing during the development process, which was basically consistent with the changes in GA content; this suggests that the gene may have a regulatory effect on flower formation (Figure 7). In addition, DELLA proteins can transmit GA signals to various regulatory pathways and mediate the expression of other flowering genes, including *PIF*, *SPL,* and *LFY*, in order to regulate flower formation. However, it is necessary to further verify whether there is a reciprocal relationship between these genes and DELLA proteins in *C. azalea* [47,48]. Moreover, while gibberellin stimulates flowering in *A. thaliana*, it inhibits it in *Malus pumila*, demonstrating its varied regulatory effects on flower formation across different plant species [49,50]. The results of this study indicate that the gibberellin content increased significantly at both the flower induction and floral organ maturation stages, and decreased significantly at anthesis. Based on these findings, it was hypothesized that high concentrations of GA may be conducive to flower induction and floral organ maturation in *C. azalea*, whereas low concentrations of GA promote flower opening.

IAAs are of significant importance in the context of plant flower development, and their function relies on the perception of signals that are converted by signal transduction pathways into complex and diverse downstream responses that regulate plant growth and development through changes in their concentration. ARF is an important action element in the IAA signal transduction pathway, which can specifically bind to the IAA response element in the promoter region of IAA response genes, activate or repress the expression of downstream genes, and then participate in the growth and development process of plants [51]. It has been reported and analyzed that ARF genes in *V. vinifera* and *Annona squamosa* were expressed at higher levels in flower buds, suggesting a possible role for these genes in regulating early flower development [52,53]. The ARF gene was found to be upregulated at the stage of floral organ maturation (HY~EP) in this study, which is consistent with the results reported above (Figure 7). This indicates that ARF may be involved in the development of *C. azalea* buds and the formation of floral organs. In *A. thaliana*, Aux/IAA functions as a transcriptional repressor of growth hormone-inducible genes and can inhibit the transcriptional regulation of ARF by forming a dimer with ARF directly through the protein [54]. This study found that Aux/IAA and ARF exhibit nearly opposite expression patterns, with the lowest levels during the predevelopment stage and the highest during anthesis. It is speculated that in *C. azalea*, this signaling pathway regulates flower formation at various developmental stages through different combinations of Aux/IAA-ARF transcriptional regulatory elements, affecting both differentiation and stability.

Furthermore, the results of the correlation analysis indicated that more ARF genes in *C. azalea* were significantly negatively correlated with changes in IAA content, which is consistent with the partial results of ARF expression in response to changes in IAA content in *M. pumila* (Figure S1). It can be postulated that ARF genes may be induced to express by growth factors [55]. However, it has been reported in the literature that low concentrations of IAA inhibit ARF activity, while a certain level of IAA concentration promotes Aux/IAA protein degradation and thus activates ARF gene expression [56]. It follows that since Aux/IAA and ARF can constitute a number of different interaction combinations, resulting in potentially different biological functions in different species.

Other endogenous plant hormones, including ABA, CTK, and BR, are also crucial to the complex regulatory system governing floral development (Figure 7 and Figure S1). ABF is a core gene in the ABA signal transduction pathway, and in this study, the ABF gene showed a significant negative correlation with ABA content, with the most significant expression at anthesis, which may be related to the regulation of the flowering process in *C. azalea*. Moreover, ABA regulates the expression of genes involved in flower formation, such as FT and CO, which consequently affects the timing of flowering in plants [22]. The involvement of CTK in the floral transition and differentiation of floral meristem cells in plants is now well established, and most of the genes located in the CTK signaling pathway in the present study also showed increased expression at the stages of flower induction and floral organ maturation [57,58]. In conclusion, it is evident that there are not only synergistic or antagonistic effects between endogenous plant hormones, but also tandem action of various floral pathways. Therefore, further experiments are necessary to verify the biological functions of key genes in each pathway.

### 3.5. Comparison of Flowering Mechanisms Between C. azalea and Most Camellia Species

*C. azalea* and winter–spring flowering Camellia species (e.g., *C. reticulata* and *C. sinensis*) are closely related yet exhibit significant differences in their flowering mechanisms, with the primary distinctions manifested in the characteristics of flower bud differentiation, responses to environmental signals, and specific expression of flowering genes. Some studies have found that *C. azalea*, *C. reticulata*, and *C. japonica* (winter–spring flowering) may all be regulated by core genes in pathways such as the photoperiod pathway and plant hormone signal transduction, including the *FT*, *PIF*, and CO genes and DELLA proteins [11,59,60]. On the other hand, the accumulation of endogenous hormones such as gibberellin, auxin, and abscisic acid plays a key role in flower development of the two flowering types of Camellia, while their concentration thresholds and the timing of expression of signal transduction-related genes may differ [61]. Therefore, *C. azalea*, *C. reticulata*, *C. japonica*, and other winter–spring flowering species have evolved unique molecular mechanisms through long-term evolution in response to seasonal changes, thereby regulating their flowering in different seasons.

Based on previous studies, most Camellia species are considered to be long-day plants and are highly sensitive to photoperiods. Even under suitable temperature conditions, if the light exposure duration is less than the critical daylength, flower buds cannot form normally [62]. This study reveals that *C. azalea* usually enters the reproductive growth stage in April and May; similarly, other winter–spring flowering species such as *C. reticulata* and *C. japonica* also form flower buds during this period. A key distinction is that *C. azalea* enters the full flowering stage around July, while species such as *C. reticulata* and *C. japonica* do not enter the full flowering stage until November. Comprehensive analysis suggests that light duration may be a key factor in promoting flower bud formation in Camellia species. In the subsequent stages of flower bud development and flowering; however, due to differences in their flowering seasons, *C. azalea* may have fundamental differences in temperature requirements compared with winter–spring flowering Camellia species (e.g., *C. reticulata* and *C. sinensis*).

Relevant studies have demonstrated that the expression level of the *CaAPX* gene in *C. azalea* is significantly upregulated under heat stress (38 °C) treatment, and the heat tolerance of plants overexpressing the *CaAPX* gene is significantly enhanced [63]. Therefore, we hypothesize that *C. azalea* can bloom normally in summer high-temperature environments, which may have developed a unique heat-tolerant flowering regulatory mechanism during the long-term evolutionary process. This study analyzed the expression profiles of key flowering genes and found that core genes in temperature and autonomous pathways, such as *FCA*, *FPA*, and *AGL*, exhibited stage-specific expression patterns. Studies have demonstrated that *FCA* is involved in both the autonomous pathway and the temperature pathway. Its transcriptional and protein levels are significantly higher at 23 °C than at 16 °C. Moreover, *FCA* induces the expression of *FT* at high temperatures to promote flowering, which indicates that the *FCA* gene plays a key role in plant responses to temperature [44,64]. Further analysis suggests that these genes jointly regulate the flowering progress of *C. azalea* by responding to high-temperature signals and integrating other flowering pathways. Conversely, species such as *C. reticulata* and *C. japonica* may be more adapted to low-temperature environments, thus flowering in winter and spring. In tea plants, the vernalization pathway gene *CsFLC1* was highly expressed in winter axillary buds (physiological dormancy period), and its transgenic plants could respond to low temperatures. It is speculated that this gene functions to inhibit vegetative growth and maintain dormancy [65]. It can, thus, be inferred that under different temperature conditions, these flowering genes exhibit specific expression patterns, thereby regulating the seasonal flowering of Camellia species.

## 4. Materials and Methods

### 4.1. Collection of Sample Materials

The experimental materials were collected from the arboretum of Southwest Forestry University in Kunming, Yunnan Province, China (25°3′ N, 102°45′ E), with a total of eight developmental periods (Figure 1). During each developmental stage, five to eight samples were collected. Three biological replicates were established for each sample, which were then segregated into PC tubes by developmental period. The samples were immediately frozen in liquid nitrogen and subsequently stored in an ultra-low-temperature refrigerator at −80 °C. Subsequently, the samples were sent to Shanghai Personalbio Technology Co., Ltd. for transcriptome sequencing. During sample collection, the external morphology and size of the flower buds at different developmental stages were observed and measured, with a sample size error margin not exceeding 0.5 mm.

### 4.2. Determination of Endogenous Hormone Content

The samples were collected in sync with the transcriptome sequencing developmental stage. These samples were frozen in liquid nitrogen immediately after collection and stored at −80 °C until extraction. Concentrations of IAA (ng/g), GA (ng/g), ABA (ng/g), CTK (ng/g), and BR (ng/g) within the samples were ascertained using the enzyme-linked immunosorbent assay (Plant ELISA Kit, Sinobestbio, Shanghai, China), following prescribed methodologies. Thereafter, the absorbance (OD value) was quantified at 450 nm with an enzyme marker (Multiskan FC, Thermo Fisher, Shanghai, China). With the set standard concentration as the x-axis and the measured OD value as the y-axis, the linear regression curve of the standard was plotted using Excel, and the endogenous hormone content in each sample was calculated in accordance with the standard curve. Three biological replicates were established for each stage.

### 4.3. RNA Extraction and Detection

At each developmental stage, 0.5–1 g of the frozen samples was weighed and ground into powder in liquid nitrogen. Total RNA was extracted separately using the EASYspin Plus Complex Plant RNA Kit (Aidlab Biotechnologies Co., Ltd., Beijing, China) following the manufacturer’s instructions. The quality of the extracted RNA was evaluated using a Nanodrop 2000 UV spectrophotometer (Thermo Fisher, Shanghai, China) and 1% agarose electrophoresis gel. RNA exhibiting a 260/280 ratio between 1.8 and 2.0, and a RIN value greater than 8.0, was selected for subsequent analyses.

### 4.4. Library Construction and Sequencing

The mRNA was isolated using Oligo (dT) magnetic beads, and then RNA was fragmented into short segments of approximately 300 bp through ionic interruption. The RNA was employed as a template for the synthesis of the first strand of cDNA, which was accomplished with the use of a 6-base random primer and reverse transcriptase. This first strand of cDNA then served as a template for synthesizing the second strand. After library construction, the fragments underwent polymerase chain reaction (PCR) amplification. This was followed by quality control using the Agilent 2100 Bioanalyzer (G2939B, Agilent, Beijing, China), which confirmed that the library met the necessary sequencing requirements. Sequencing was performed using the Next-Generation Sequencing (NGS) technique on the Illumina HiSeq platform (Illumina, San Diego, CA, USA). Sequencing was conducted on the Illumina HiSeq sequencing platform.

### 4.5. Differential Expressed Genes (DEGs) Screening and Enrichment Analysis

The transcript sequence served as a reference for comparing the Clean Reads of each sample to the reference. This was conducted using RSEM (RNA-Seq by Expectation-Maximization, v1.3.1) expression quantification software, with the FPKM value of each gene subsequently calculated. DESeq was used to analyze differential gene expression across various developmental periods. Differentially expressed genes were identified using the following criteria: log2FoldChange > 1 and *p*-value < 0.05. In order to identify the functional pathways significantly enriched in differentially expressed genes, both Gene Ontology (GO, https://geneontology.org/, accessed on 7 April 2024) and Kyoto Encyclopedia of Genes and Genomes (KEGG, https://www.genome.jp/kegg/, accessed on 7 April 2024) enrichment analyses used a *p*-value < 0.05 as the criterion for significant enrichment. The top 30 and 20 pathways with the most significant enrichment were selected for statistical mapping, respectively.

### 4.6. Real-Time PCR Validation

In this study, the expression of genes related to flower formation in *C. azalea* was analyzed. Several genes that exhibited significant differential expression were selected for validation of the transcriptome data. Firstly, RNA from each of the eight developmental periods of *C. azalea* was subjected to first-strand cDNA synthesis (All-In-One 5 × RT MasterMix, abm). Primer sequences were then designed using the Meinverse software (https://meinverse.cn, accessed on 23 August 2024) according to the principle of fluorescent quantitative primer design and sent to Tsingke Biotech Co., Ltd. (Kunming, China). for synthesis (Primer Information Table S2). The reaction system was as follows: BlasTaq^TM^2 × qPCR 10 μL, forward primer 0.5 μL, reverse primer 0.5 μL, Template DNA 1 μL, nuclease-free H_2_O 8 μL. The reaction procedure was as follows: Enzyme Activation 95 °C for 3 min, Denaturation 95 °C for 15 s, Annealing/Extension 60 °C for 1 min, 40 cycles (Blastaq^TM^2 × qPCR Mastermix, abm). The EFIα gene was employed as an internal reference, with three biological replicates for each gene. The data were expressed as mean ± standard error. The relative expression of the genes was calculated using the 2^−ΔΔCt^ method. Finally, the data were organized using Excel and plotted using Origin 2022.

## 5. Conclusions

In summary, based on external morphological observations and transcriptome analysis, this study divided the eight developmental stages of *C. azalea* into three growth stages: floral induction, floral organ maturation, and anthesis. Through the comparative analysis of representative samples from each growth stage of *C. azalea*, we found that differentially expressed genes (DEGs) are primarily involved in pathways such as the specification of plant organ identity, plant hormone signal transduction, and photosynthesis. Additionally, we hypothesized that photoperiod is a key pathway inducing floral induction, while core genes in the temperature pathway and autonomous pathway—such as *FCA* and *AGL*—may co-regulate the summer flowering of *C. azalea* by integrating high-temperature signals with other flowering pathways. Furthermore, ABA, CTK, GA, and BR accumulate stably during the floral organ maturation stage of *C. azalea* and decrease significantly after anthesis, whereas IAA exhibits the opposite trend. These results indicate that ABA, CTK, GA, and BR may be important endogenous hormones affecting floral induction and floral organ maturation in *C. azalea*, whereas IAA may be a key factor determining its anthesis. To gain a deeper understanding of the unique regulatory mechanisms of flower formation in *C. azalea*, further investigation into the interactions between endogenous hormones and the flowering pathways, as well as the spatial and temporal crosstalk among multiple hormones, is necessary.

## Data Availability

The original contributions presented in this study are included in the article/Supplementary Material. Further inquiries can be directed to the corresponding author.

## Supplementary Materials

The following supporting information can be downloaded at https://www.mdpi.com/article/10.3390/plants14152291/s1.
