# Supplementary material for: Transcriptome and Endogenous Hormones Reveal the Regulatory Mechanism of Flower Development in Camellia azalea
Source: Plants (Basel). 2025 Jul 25;14(15):2291. doi: 10.3390/plants14152291 (PMC12348097; doi:10.3390/plants14152291)
Supplement: Supplementary file 1 [file plants-14-02291-s001.zip › plants-3726621-supplementary.pdf]

Supplementary materials

Table S1. Raw transcriptome sequencing data of *Camellia azalea* samples

Sample	Raw Reads	Bases (bp)	N (%)	Q20 (%)	Q30 (%)
YY1	38108982	5754456282	0.000440	97.82	93.80
YY2	39256558	5927740258	0.000439	97.67	93.48
YY3	42691458	6446410158	0.000446	97.83	93.81
ZY1	36741474	5547962574	0.000440	97.92	93.96
ZY2	36245436	5473060836	0.000443	97.80	93.70
ZY3	38069598	5748509298	0.000449	97.94	94.02
HY1	39786674	6007787774	0.000325	98.19	94.56
HY2	36569712	5522026512	0.000329	97.98	94.07
HY3	38205504	5769031104	0.000331	98.18	94.59
HB1	39548156	5971771556	0.000324	98.03	94.20
HB2	35856438	5414322138	0.000324	98.05	94.24
HB3	45421456	6858639856	0.000370	98.08	94.30
EP1	37448138	5654668838	0.000330	98.04	94.21
EP2	40180154	6067203254	0.000330	98.08	94.32
EP3	38808010	5860009510	0.000323	98.22	94.68
LS1	45288834	6838613934	0.000366	98.01	94.10
LS2	39987922	6038176222	0.000331	97.94	93.98
LS3	42623202	6436103502	0.000321	97.98	94.08
BKF1	40547042	6122603342	0.000322	98.00	94.25
BKF2	38231486	5772954386	0.000327	97.79	93.77
BKF3	40157876	6063839276	0.000327	98.02	94.28
SK1	39544004	5971144604	0.000324	97.71	93.66
SK2	45597814	6885269914	0.000367	97.84	93.85
SK3	46119644	6964066244	0.000376	97.88	93.00

Table S2. DEGs enriched in the specification of floral organ identity pathway

Gene ID	Annotation
c11361_g1	Axial regulator YABBY 1
c18279_g1	Axial regulator YABBY 5
c15184_g1	Agamous-like MADS-box protein MADS2
c16000_g1	Agamous-like MADS-box protein MADS2
c16423_g1	Agamous-like MADS-box protein MADS2
c16525_g1	Agamous-like MADS-box protein MADS2
c16143_g1	Agamous-like MADS-box protein MADS3
c16810_g1	Agamous-like MADS-box protein MADS3
c19143_g1	Agamous-like MADS-box protein MADS3
c15251_g1	Agamous-like MADS-box protein MADS4
c15802_g1	Agamous-like MADS-box protein MADS4
c16195_g1	Agamous-like MADS-box protein MADS4
c6353_g1	Agamous-like MADS-box protein MADS4
c17774_g1	Agamous-like MADS-box protein MADS9
c18534_g1	Agamous-like MADS-box protein MADS9
c15217_g1	Agamous-like MADS-box protein TM6
c17620_g1	Agamous-like MADS-box protein TM6
c18497_g1	Agamous-like MADS-box protein TM6
c18876_g1	Agamous-like MADS-box protein TM6
c19755_g1	Agamous-like MADS-box protein TM6
c16564_g1	Agamous-like MADS-box protein AGL8 homolog
c14475_g1	Agamous-like MADS-box protein AP1
c16698_g1	Agamous-like MADS-box protein AP1
c4561_g1	Floral homeotic protein APETALA 2
c5837_g1	Floral homeotic protein APETALA 2
c16360_g1	APETALA2-like protein 1
c3353_g1	APETALA2-like protein 1
c14452_g1	WUSCHEL-related homeobox 8

Table S3. The primers sequences of candidate genes used in qRT-PCR experiment

Gene Name	Forward primer,	Reverse primer
c4280_g1（CTK）	GTTAAGCGGATTTCCCGTGG	AAACACGAGGTTGGGCATCT
c5570_g1（BR）	AAACCGCCAATGTTCAGTCC	TTTGTGGCAGCTCTCAACTC
c5837_g1（AP2）	AATTTTCCGAGGGCTCACTG	AAAGCTCCACCGATTTTCCG
c8308_g1（IAA）	TTGGCTTCTTCGGCTTCCAT	CCGAGCTAAGCAAGTGGAGT
c9396_g1（ABA）	TGTGTTGCAGAGTTGTTGGC	TCAACTGCTTTTCCGCACTC
c10643_g1（CO）	TATCACCAGCACGAAACCAC	ATTTCATCTCCGGCTCGTTG
c11361_g1（YABBY）	TAAGTCACAGGGAGGCTTTCAG	ACGGGTTGATCAGGCAAAAG
c16143_g1（MADS3）	GGAGAGGTACCAACATTGCAAC	TGCGTTGAAGGGATTCGAAC
c16698_g1（AP1）	ACTTCCCTGCCTCAACATTG	AGCAAAGCATCCAAGGTCAC
c18787_g1（SPL）	AACGTTTCTGCCAGCAATGC	GCCTGATCCTTCTCCATAAGACTC
c18876_g1（AGL）	ATCGACCTATGGAGCACACAC	TTTTGCTCAAGACCGCACAG
c19122_g1（ELF）	TGCGAGAAGGGTTGTGGATC	TTCTGGCCACCCTTTCCATC
c19689_g1（GA）	CCTCTTCAAACCCTCCTCTTCTTC	ACCTCATCTTTTCCCCAACGAG
c19862_g1（FLC）	ACTTCAGACACCCGTTTTGC	CTTGGGACTCCAGCTCATATTG
c20448_g1（IAA）	GCCGCGATGAGAAGGAAAAG	TCTTTTGTTGCGTGGCGTTG

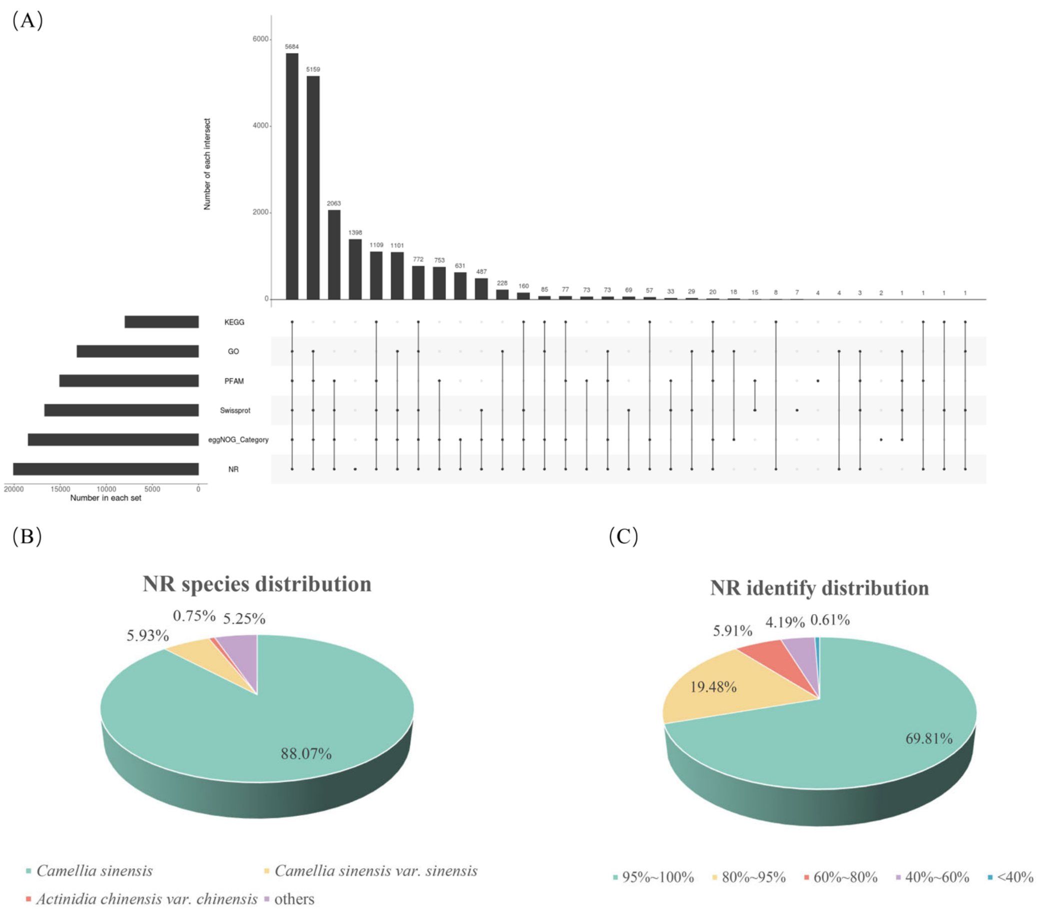

Figure S1. Assembly of C. azalea Unigenes annotations. (A) Upset plots of C. azalea Unigenes anno-tated in different databases. The number in each set indicates the number of Unigenes annotated in each database; the number in each intersection indicates the number of common Unigenes an-notated in multiple databases. (B) Species matching annotation diagram of C. azalea Unigenes. (C) Annotated diagram of homologous sequence matches for C. azalea Unigenes.

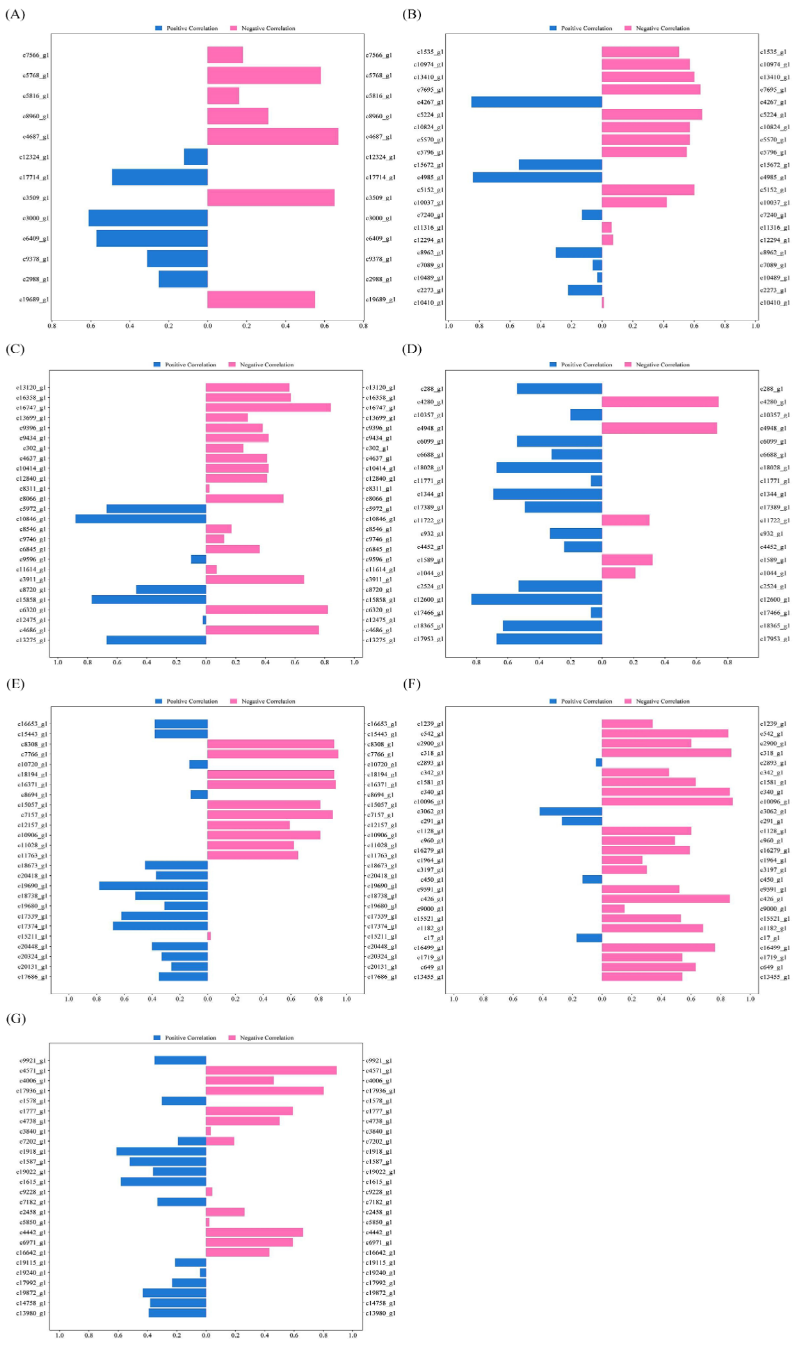

Figure S2. Correlation analysis between hormone levels and associated genes. A represents the correlation analysis between GA levels and associated genes. B represents the correlation analysis between BR levels and associated genes. C represents the correlation analysis between ABA levels and associated genes. D represents the correlation analysis between CTK levels and associated genes. E represents the correlation analysis between IAA levels and Auxin-responsive protein IAA. F represents the correlation analysis between IAA levels and Auxin response factor. G represents the correlation analysis between IAA levels and other associated genes of IAA.
